# Plasmonic metalens based on coupled resonators for focusing of surface plasmons

**DOI:** 10.1038/srep37861

**Published:** 2016-11-29

**Authors:** Quan Xu, Xueqian Zhang, Yuehong Xu, Quan Li, Yanfeng Li, Chunmei Ouyang, Zhen Tian, Jianqiang Gu, Wentao Zhang, Xixiang Zhang, Jiaguang Han, Weili Zhang

**Affiliations:** 1 Center for Terahertz waves and College of Precision Instrument and Optoelectronics Engineering, Tianjin University and the Key Laboratory of Optoelectronics Information and Technology (Ministry of Education), Tianjin 300072, China; 2 Cooperative Innovation Center of Terahertz Science, Chengdu 610054, China; 3 College of Electrical Engineering and Automation, Guilin University of Electronic Technology, Guilin 541000, China; 4 Divison of Physical Science and Engineering, King Abdullah University of Science and Technology, Thuwal 23955-6900, Saudi Arabia; 5 School of Electrical and Computer Engineering, Oklahoma State University, Stillwater, Oklahoma 74078, USA

## Abstract

As an essential functionality, flexible focusing of surface plasmons (SPs) is of particular interest in nonlinear optics and highly integrated plasmonic circuitry. Here, we developed a versatile plasmonic metalens, a metasurface comprised of coupled subwavelength resonators, whose optical responses exhibit a remarkable feature of electromagnetically induced transparency (EIT). We demonstrate numerically and experimentally how a proper spatial design of the unit elements steers SPs to arbitrary foci based on the holographic principles. More specifically, we show how to control the interaction between the constituent EIT resonators to efficiently manipulate the focusing intensity of SPs. We also demonstrated that the proposed metalens is capable of achieving frequency division multiplexing. The power and simplicity of the proposed design would offer promising opportunities for practical plasmonic devices.

Surface plasmons (SPs) are a special electromagnetic surface wave that is confined to propagate at the metal-dielectric interface with an exponential decay in both directions perpendicular to the interface^1^. Owing to such a unique property, SPs offer a remarkable two-dimensional (2D) optical platform on which electromagnetic waves can be manipulated at a subwavelength scale. This renders SPs very attractive in developing next-generation, ultra-compact, ultra-fast integrated plasmonic circuitry in which light and electric signals can be transferred and processed simultaneously^2^,^3^,^4^. In the last decade, with the development of subwavelength optics and fabrication technologies, manipulating SPs by artificial structured surfaces (metasurfaces) has garnered a lot of attention^5^,^6^,^7^. For instance, a series of SP-based applications have been demonstrated by metasurfaces, including SP metalenses^8^,^9^,^10^,^11^, unidirectional launchers^12^,^13^,^14^,^15^, wavelength demultiplexers^16^,^17^,^18^,^19^,^20^, special SP beams^21^,^22^,^23^,^24^,^25^ and SPs wavefront engineering^26^. Among these, metalenses have been intensively explored in recent years since they can couple free-space electromagnetic waves to SPs and then focus them to spatial regions that are much smaller than the diffraction limit. This results in a substantial improvement in the electric intensity, enabling applications in areas, such as nonlinear optics^27^ and nanoscale waveguides^28^,^29^.

SP focusing is normally based on optical interference, where the constructive interference in the superposition of SPs with phase matching at a designated spot can lead to a considerable enhancement in the electric field. In early studies, design of SP focusing was generally achieved by controlling the metallic geometry, such as semicircular slits filled with dielectric media^30^, symmetry-broken corrals^31^, or Archimedes’ spiral slits with circularly polarized incidence^32^. In these studies, the focusing spot was normally located at the center of the structure, which limits further practical applications. In addition, only a single ring-shaped structure was adopted, which limited the focusing intensity due to the lack of coupling section. To improve this situation, subwavelength bar-shaped slit resonator (BSR) made from metal film was commonly used in recently works^9^,^10^,^11^,^12^,^13^,^17^,^26^,^33^. By applying holographic principles^9^, iterative algorithm^17^, or geometric phase concept^26^, the location of focus can be pre-designated, and meanwhile the focusing intensity is also enhanced since the effective coupling section is increased. Most importantly, the coupling conversion efficiency that from the free space light to SPs is improved by optimizing the BSR geometry taking advantage of plasmonic resonance. As the most straightforward method, if the BSRs are appropriately placed based on the holographic principle^9^, where the locations of the BSRs are carefully chosen to match the propagation phase of all excited SPs to be equally superposed at a designated spot and if all the SPs are excited in phase, constructive interference occurs and a conventional SP focusing lens is achieved.

However, for an individual BSR, it selectively scatters incident light polarized perpendicular to its long axis, giving rise to SPs which propagate radially away from the both sides of the BSR with initial phase difference π^13^. For the holographic metalens based on BSRs, only the SPs on one side of BSRs will contribute to the focusing spot, meanwhile the SPs on the other side will diverge away^9^. Particularly, focusing along the direction perpendicular to the incidence polarization is hard to be achieved. Furthermore, although the intensity of the focus can be manipulated by adjusting the number of BSRs, the contribution of each BSR is dependent on the relative location between the BSR and focus. This means that accurate manipulation of the intensity of focus is complicated because the contribution of each adjusted slit needs to be calculated and the sum of all such contributions ought to be exactly equal to the desired manipulation. These problems are unavoidable if only one kind of slit is used in the design. Recently, by taking advantages of the electromagnetically induced transparency (EIT) effect in metamaterials^34^,^35^,^36^, we preliminarily demonstrated that the SPs excited from the coupled slit pair (EIT-pair) can be effectively controlled through manipulating the near-field coupling between the constituents^37^. However, such a concept is believed to enable more promising applications, especially in practical plasmonic device designs, such as focusing lens. Continued interest in this subject is fueled and extended, and thus here we reported on one strategy to create a resonant plasmonic metalens with versatile functionalities based on the EIT effect. A detailed theoretical and experimental study were implemented in this work to show that the proposed metalens not only can manipulate the location and intensity of SP focusing at will, but also can be used for frequency division multiplexing (FDM). Our proposal may provide a desirable focusing method and operation for SPs in the broad spectrum of electromagnetic waves.

## Results

### Metalens based on single kind of slit resonators

**Figure 1:**
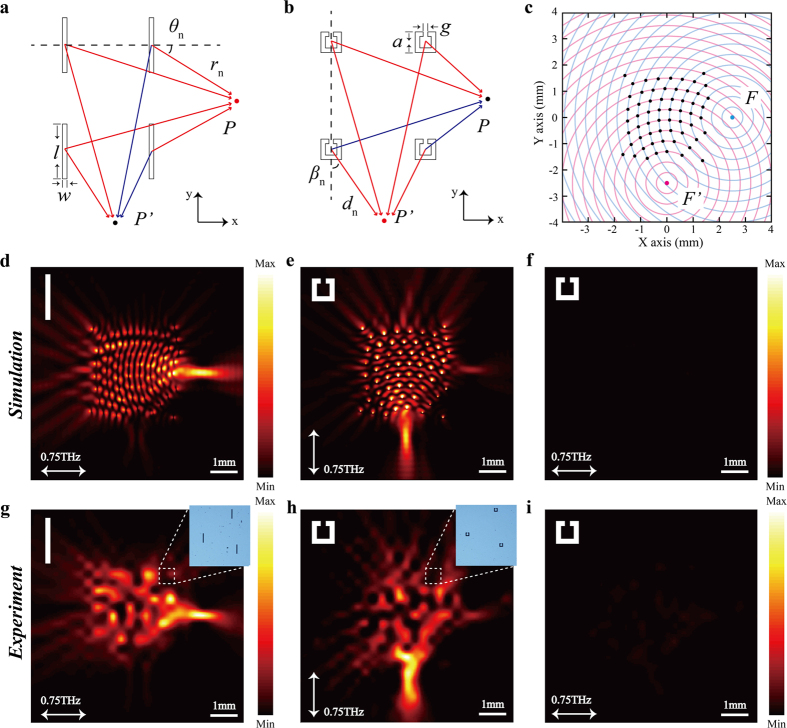
Holographic metalens designs based on BSR and SSR. (**a,b**) Schematic views of the calculation based on the 2D Huygens-Fresnel principle. The geometric parameters are: *l* = 120 μm, *w* = 10 μm, *a* = 45 μm, *g* = 10 μm. (**c**) Schematic view of the metalens design based on holographic principles; the center of the blue circles is *F* (2500, 0), and the center of the red circles is *F’* (0, −2500). (**d–f**) Simulated results of the intensity profiles (|*E*_*Z*_|^2^) at 0.75 THz obtained at 50 μm above the metalens. The inset in the top left corner of each figure schematically shows the shape of the unit element, and the incident polarization is indicated by the corresponding white arrow, similarly hereinafter. (**h–g**) Experimental results of the intensity profiles corresponding to (**d–f**), respectively. The microscopic images of part of the fabricated metalenses are shown as insets.

Considering *n* BSRs are located at different positions, as shown in Fig. 1a. At the point of observation, *P* (one point located on the right side of BSRs), the complex amplitude of the electric field *E*_P_ is the superposition of all propagating SPs arriving at *P*^17^,^38^. Since all BSRs are located on the left side of *P*, only the SPs propagating in the +*x* direction contribute to the electric field. If we assume that *r*_*n*_, the distance between the *n*-th BSR and *P*, is equal to an integral multiple of *λ*_*Sp*_ (wavelength of designated SPs) and because all the +*x* direction propagating SPs have the same initial phase, the SPs must interfere constructively arriving at *P*. Similarly, for a point *P’* located on the bottom side, the complex amplitude of the electric field *E*_P’_ is the superposition of all arrived SPs. However, the propagating SPs toward the −*x* and +*x* direction will arrive at *P’* with an initial phase difference π, where the out-of-phase SPs tend to diminish each other. When considering *n* split-ring-shaped slit resonators (SSRs) are located at different positions, as shown in Fig. 1b. As expected, the SSR can only be excited under the *y*-polarized incident beam and most of the excited SPs propagate along ±*y* direction^37^. Therefore, the SPs excited from SSRs will constructively interfere at *P’* and destructively interfere at *P*.

As a typical example to verify the above analysis, we concentrate on a dual-focus metalens with orthogonal focusing directions. As shown in Fig. 1c, if we assume that there are two imaginary emitters placed at *F* (2500, 0) and *F’* (0, −2500) where SP focusing is desired, the wavefronts of the SPs propagating out from the imaginary emitters can be defined by the blue and red concentric circles, respectively. Because metals (Aluminum is chosen in this work) exhibit large electrical conductivities with *ε_m_* ≫ 1 in the THz regime, the dispersion relation of THz SP is very close to that of the free-space wave, namely 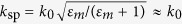, where *k*_sp_ and *k*_0_ are the wavenumbers of the SPs and free-space light, respectively^39^,^40^. Here, the radial difference between the neighboring concentric circles is 400 μm according to 0.75 THz, and the unit elements are placed at the intersections of these circles (60 unit elements approximately occupy an area of 3.2 × 3.2 mm). Because the unit elements lie on the equiphase lines (wavefronts of the SPs), the excited SPs will thus constructively interfere at the *F* and *F’* if they are excited in phase.

To illustrate the SPs behavior in the proposed design, we ran computer simulations on spectral response and electric field distributions using the commercial software *CST Microwave Studio* (see Methods). Figure 1d shows the simulated intensity profiles of the BSR-based mentlens according to the holographic design in Fig. 1c. It is evident that launching and focusing at *F* are possible; however, only a single focus can be obtained. The SP excitation efficiency of the BSR based metalens is estimated to be 3.66% by coating a 20 μm thick dielectric film on the metasurface^26^. Similarly, only a single focus at *F’* can be obtained in SSR-based metalens under *y*-polarization incidence (as shown in Fig. 1e). Furthermore, since the SSRs cannot be excited under *x*-polarization incidence, the simulated intensity profiles at *F* and *F’* in Fig. 1f are both quite weak. Therefore, due to the dipole responses of the BSR and SSR, the focusing along the direction perpendicular to the incident polarization cannot be obtained if only one kind of resonators is adopted.

### Metalens based on EIT-pair

**Figure 2:**
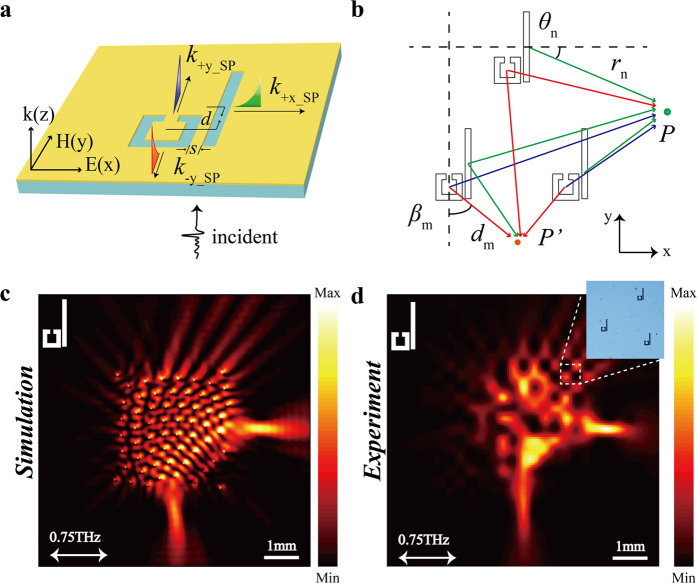
EIT-pair based metalens design. (**a**) Schematic view of EIT-pair, the geometric parameters are: *d* = −40 μm, *s* = 5 μm. (**b**) Schematic views of the calculation based on the 2D Huygens-Fresnel principle. (**c,d**) Simulated and measured results of the intensity profiles (|*E*_*Z*_|^2^) at 0.75 THz obtained at 50 μm above the metalens, respectively.

To acquire the obvious EIT effect, previous metamaterial system is generally composed of two artificial resonant elements, a radiative bright one that strongly couples with the light in the free space and a dark one that weakly couples to the incident light^34^,^35^,^36^. It has been demonstrated that asymmetric excitation of SPs can be achieved by EIT-pair, where the BSR and SSR act as the bright and dark elements, respectively^37^. As shown in Fig. 2a, the SPs are excited and then propagate along the ±*y* direction and +*x* direction; meanwhile the SPs that propagate in the −*x* direction are strongly suppressed. By applying the coupled Lorentzian oscillator model, it can be deduced that the ±*y*-direction propagating SPs have a ±π/2 phase difference with that along the +*x* direction^37^. Note that the SPs propagating in the ±*y*-direction operate with a narrowband due to the EIT effect. Nonetheless, the holographic scheme usually ideally works for a single wavelength, thus it is feasible to use the EIT-pair for holographic metalens design with a certain target wavelength.

Considering *n* EIT-pairs are located at different positions, as shown in Fig. 2b. At point *P*, the SPs propagating along ±*y* directions tend to diminish each other due to destructively interference. Meanwhile, the SPs propagating in the +*x* direction constructively interfere and then results in SPs focusing. At point *P’*, the SPs propagating in the −*y* direction constructively interfere, and all the SPs propagating in the +*x* direction excited by the EIT-pairs located on the left side also constructively interfere (with a π/2 phase difference between these two parts of SPs). The simulated intensity profile of EIT-pair based metalens under the *x*-polarization incidence are shown in Fig. 2c, where the simulated electric-field intensity at *F* and *F’* are extremely strong. Note that the left towards diverging SPs of BSR based metalens (see Fig. 1d), are converted to the ±*y* directions when taking EIT-pair as the element, wherein the SPs along the −*y* direction contribute to the focusing spot *F’*. In this case, the conversion efficiencies from the excited SPs to the focusing spots of the BSR and EIT-pair based metalens are estimated to be 16.51% and 23.92%, respectively, by coating a 20 μm thick dielectric film on the metasurface^26^. Clearly, using EIT-pair for holographic design could further utilize the energy.

**Figure 3:**
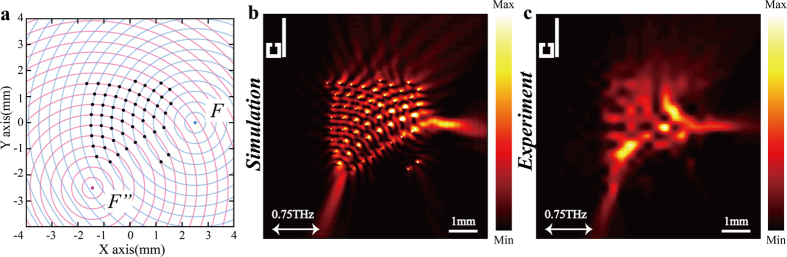
Another EIT-pair based metalens design. (**a**) Schematic view of the metalens design based on holographic principles; the center of the blue circles is *F* (2500, 0), and the center of the red circles is *F”* (−1250, −2500). (**b,c**) Simulated and measured results of the intensity profiles (|*E*_*Z*_|^2^) at 0.75 THz obtained at 50 μm above the metalens, respectively.

To experimentally demonstrate the proposed metalens, we fabricated the samples with 200-nm-thick aluminum structures on a 2-mm-thick quartz substrate by use of conventional photolithography. To map the electric-field intensity profiles on top of the sample, a near-field terahertz microscope system we recently developed was applied to scan the SP field^41^. The measured intensity profiles at 0.75 THz, as illustrated in Figs 1 and 2 agree well with numerical simulations. This good agreement experimentally confirms that in the proposed metalens design, focusing along the direction perpendicular to incident polarization cannot be achieved by adopting single kind of slit resonator as the unit element. Nonetheless, a dual-focus metalens can be obtained based on the EIT-pair consisting of both the BSR and SSR. Since the proposed method could provide the flexibility to manipulate the focusing locations, another metalens design is illustrated in Fig. 3a, where the foci are respectively located at *F* (2500, 0) and *F”* (−1250, −2500) and the radial difference between the neighboring concentric circles is also 400 μm (53 EIT-pairs approximately occupy an area of 3.2 × 3.2 mm). The simulated and experimental intensity profiles as shown in Fig. 3b and c agree well with each other, which indicate that the proposed method provides versatile capabilities to manipulate various focusing locations.

### Manipulation of focusing intensity

**Figure 4:**
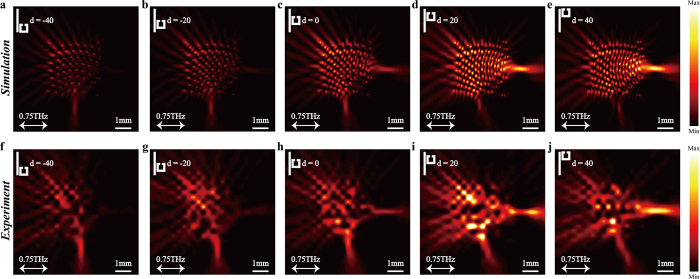
Electric field intensity profiles for different *d*. (**a–e**) Simulated results of the intensity profiles (|*E*_*Z*_|^2^) at 0.75 THz obtained at 50 μm above the metalens with *d* varying from −40 to 40 μm and *s* = 5 μm at 0.75 THz, respectively. (**f–j**) Measured results of the intensity profiles corresponding to (**f–j**), respectively.

What more intriguing is that this EIT-based metalens allows the manipulation of focusing intensity with geometrically tailored constituent elements. The essence of the manipulation strategy is the same as that in most typical EIT metamaterials^42^,^43^, where the coupling can be simply changed by varying the geometric position, such as the relative location *d* between the bright and dark elements. In the preceding analysis, the intensity at *F* is mainly dominated by the SPs propagating in the +*x* direction. If we placed the SSRs at the right side of BSRs with *d* = −40 μm, as shown in Fig. 4a, strong coupling occurs between the resonators. Consequently most of the SPs propagating in the +*x* direction are converted to the ±*y* direction with opposite phase, resulting in diminished intensity of the electric field at *F*. As *d* changes from −40 to 40 μm, the coupling between the SSRs and BSRs becomes weaker, and then the uncoupling part of the SPs propagating in the +*x* direction will be enhanced and constructively interfere at *F*. The numerically simulated and experimentally measured intensity profiles of the metalens when *d* changes from −40 to 40 μm are illustrated in Fig. 4. Clearly, the resulting intensity at the focus point, *F*, is enhanced as *d* gradually increases from −40 to 40 μm.

**Figure 5:**
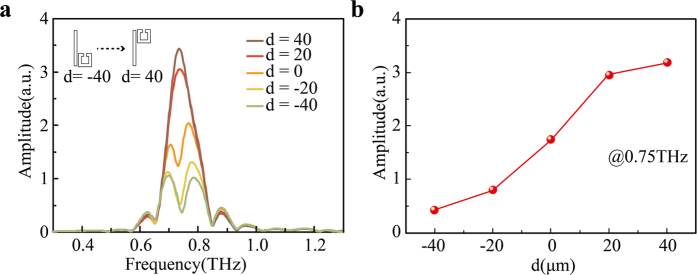
Variation in simulated SP amplitude spectra with respect to *d*. (**a**) Simulated SP amplitude spectra at *F* with *d* varying from −40 to 40 μm. (**b**) Variation of the SP amplitude at 0.75 THz at *F* with respect to *d*.

The observed variation of the SP strength at *F* can also be clearly seen in the corresponding spectral response. Figure 5a shows the changes of the SP amplitude spectra with respect to *d* at *F*. At *d* = −40 μm, a remarkable resonance dip is observed at 0.75 THz. When the distance *d* varies from −40 to 40 μm, the corresponding resonance dip undergoes a strong modulation. When *d* = 40 μm, the resonance dip completely disappears and only a single resonance peak is observed in the SPs spectra. Such a variation from a dip to a peak is also clearly seen in Fig. 5b, where the simulated electric field amplitude at 0.75 THz at focus, *F*, is increased monotonically as a function of *d*. It means that accurate manipulation of the focusing intensity can be achieved by carefully choosing *d*. Note that the design strategy of intensity manipulation here can be achieved not only by varying *d*, but also any other geometric parameters that can change the coupling coefficient. For any holographic design, it is desired to have reconfigurable hologram^44^. Here the proposed design possesses the promising possibility for reconfigurable focusing spot, since the EIT effect could be externally controlled by integrating tunable materials as part of the unit elements^35^,^45^,^46^, or directly varying the relative geometric positions using microelectromechanical system^47^,^48^.

### Dual-frequency metalens

**Figure 6:**
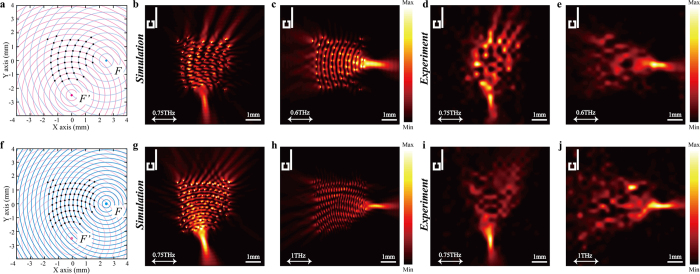
Metalens designed for FDM based on the EIT-pairs. (**a,f**) Schematic view of the metalens design. The center of the blue circles is *F* (0, −2500), corresponding to 0.75 THz; the center of the red circles is *F’* (2500, 0), corresponding to 0.6 THz in (**a**) and 1 THz in (f). (**b–e**) Simulated and measured results of the intensity profiles (|*E*_*Z*_|^2^) of the metalens designed in (**a**), respectively. (**g–j**) Simulated and measured results of the intensity profiles (|*E*_*Z*_|^2^) of the metalens designed in (**f**), respectively.

It has been demonstrated that the BSRs can also be used for FDM^9^,^17^. So far, our concern is at 0.75 THz, but in fact, according to the holographic principles, if the imaginary emitters launch SPs at different frequencies and the EIT-pairs are placed at the intersections of these wavefronts, the SPs excited by the EIT-pairs will focus at the designated spot with the corresponding frequency. As a demonstration, two dual-frequency metalenses designed by the holographic principles are shown in Fig. 6a and f, where the radial difference between the neighboring blue circles is 500 μm in Fig. 6a and 300 μm in Fig. 6f (respectively corresponding to 0.6 THz and 1 THz). The radial difference between the neighboring red circles is 400 μm in both instances (corresponding to 0.75 THz). There are 48 and 80 EIT-pairs placed at the intersections of the circles in Fig. 6a and f, respectively, approximately occupying an area of 3.2 × 3.2 mm. The designated focusing spots are at *F* (2500, 0) and *F’* (0, −2500). The simulated intensity profiles are shown in Fig. 6b,c,g and h, respectively. The measurement results illustrated in Fig. 6d,e,i and j agree well with the simulations, indicating that the EIT-pair based metalens has the ability to steer SPs at a designated focusing spot based on its frequency. Such a device is particularly attractive in constructing ultra-fast plasmonic communication systems, where FDM could play an important role in solving the strict requirements of frequency separation.

## Conclusion

We demonstrated a resonant plasmonic metalens with flexible functionalities for the SP focusing. Such a plasmonic device is driven by an EIT-pair consisting of two kinds of resonators that are essentially coupled with each other. The substantial coupling between these plasmonic elements enables a powerful metalens and enhances the launching and control of the SP focusing. The proposed design strategy is demonstrated by the successful implementation of a typical example, dual-focus metalens of various focusing positions, intensities and frequencies. By using the near-field scanning terahertz microscopy, we directly mapped the SPs and experimentally verified the design at terahertz frequencies. Since the coupling feature of the plasmonic resonators is general, the proposed concept can be easily extended to higher frequencies, such as infrared. It is clear that the plasmonic meta-atom interaction is endowed with controllable characteristics that suggest new functionalities and new possibilities in developing novel plasmonic devices.

## Methods

### Simulation

The simulations were carried out using the commercial software package *CST Microwave Studio*. The entire simulation areas are 7.8 mm × 7.8 mm. The metal in the simulations was aluminum loaded from the CST material library, and the substrate was quartz defined as *ε* = 3.76. Open boundary conditions were applied in both the *x* and *y* directions. The incident broadband plane wave was normally incident onto the structure from the substrate side. The SP spectra were extracted by setting field probes at the corresponding positions, while the field distributions of the SPs were mapped by defining the electric field monitors of *E*_z_. The simulation results were obtained at 50 μm above the metalens on the air side.

### Experiments

All the samples were characterized by a terahertz time-domain near-field scanning system. Different from the traditional terahertz time-domain spectroscopy, the terahertz detector here was a near-field photoconductive antenna-based probe (Protemics GmbH). To enable a movable probe, the detection beam of the system was coupled to a 2-m-long optical fiber. A pre-dispersion compensation grating pair was employed in the optical path to suppress pulse stretching in the fiber. The probe was fixed on a 2D electrically-controlled translation stage. In the measurements, the terahertz probe was placed approximately 50 μm above the sample surface. The 2D scanning ranges were the same as those used in the simulations.

## Additional Information

**How to cite this article**: Xu, Q. *et al*. Plasmonic metalens based on coupled resonators for focusing of surface plasmons. *Sci. Rep*. **6**, 37861; doi: 10.1038/srep37861 (2016).

**Publisher's note:** Springer Nature remains neutral with regard to jurisdictional claims in published maps and institutional affiliations.
